# The associations of physical activity, sedentary time, and sleep with V˙O_2max_ in trained and untrained children and adolescents: A novel five-part compositional analysis

**DOI:** 10.1371/journal.pone.0275557

**Published:** 2023-03-08

**Authors:** Adam Runacres, Kelly A. MacKintosh, Sebastien Chastin, Melitta A. McNarry

**Affiliations:** 1 Applied Sports, Technology, Exercise and Medicine Research Centre, Department of Sport and Exercise Sciences, Swansea University, Swansea, United Kingdom; 2 Institute of Sport, Manchester Metropolitan University, Manchester, United Kingdom; 3 School of Health and Life Sciences, Glasgow Caledonian University, Glasgow, United Kingdom; 4 Department of Movement and Sports Sciences, Ghent University, Ghent, Belgium; Universiti Malaya, MALAYSIA

## Abstract

The benefits of physical activity (PA) and the negative impacts of sedentary time (SED) on both short- and long-term health in youth are well established. However, uncertainty remains about how PA and SED jointly influence maximal oxygen uptake ($\dot{V}O_{2\max}$). Therefore, the aim of this study was to determine the joint influence of PA and SED on $\dot{V}O_{2\max}$ using compositional analyses. 176 adolescents (84 girls, 13.8 ± 1.8 years) completed an incremental ramp test and supramaximal validation bout on a cycle ergometer, with PA and SED recorded for seven consecutive days on the right hip using a ActiGraph GT3X accelerometer. Time spent in Sleep, SED, light, moderate and vigorous PA was analysed using a compositional linear regression model. Compositions with 10 minutes more time in vigorous PA (> 27.5 mins⋅day^-1^) compared to the average 17.5 mins⋅day^-1^ were associated with a + 2.9% - 11.1% higher absolute and scaled $\dot{V}O_{2\max}$ whilst compositions with less (> -10 mins⋅day^-1^) VPA were associated with a reduced absolute and allometrically scaled $\dot{V}O_{2\max}$ (-4.6% - 24.4%). All associations were irrespective of sex, maturity, and training status. The proportion of time spent sedentary had little impact on absolute and scaled $\dot{V}O_{2\max}$ (0.01–1.98%). These findings therefore highlight that intensity of PA may be of greater importance for increases in $\dot{V}O_{2\max}$ than reductions in SED and should be incorporated into future intervention designs.

## 1. Introduction

Poor maximal oxygen uptake ($\dot{V}O_{2\max}$) has been associated with an increased risk of cardiovascular and metabolic disease, leading to an increased likelihood of premature mortality across the lifespan [1–4]. $\dot{V}O_{2\max}$, defined as the highest rate of oxygen consumption despite further increases in work rate [5], is key to athletic performance, with youth athletes consistently reported to have a greater $\dot{V}O_{2\max}$ than their untrained counterparts [6–9]. However, the term $\dot{V}O_{2\max}$ in the paediatric literature is contentious as only 20–40% of children and adolescents display a plateau, and thus a supramaximal validation bout is necessary to validate a maximal effort [8, 9]. Consequently, the term peak $\dot{V}O_{2}$ will be used within this study where a supramaximal bout was not undertaken and $\dot{V}O_{2\max}$ used only when a scientifically rigorous supramaximal validation bout was employed. Whilst training is well-established to improve $\dot{V}O_{2\max}$ in youth athletes [6, 9–11], what remains less clear is the influence of habitual physical activity (PA) and sedentary time (SED) on $\dot{V}O_{2\max}$. More specifically, some studies have reported a significant association between PA and peak $\dot{V}O_{2}$ [12–14], whereas others argue that children and adolescents rarely experience PA of a sufficient duration and intensity to significantly influence peak $\dot{V}O_{2}$ [11, 15].

Moderate-to-vigorous PA (MVPA) is perhaps the most widely used PA metric in children and adolescents [16–18] and remains the focus of governmental physical activity guidelines [19, 20]. Research in children has consistently shown that increased levels of MVPA are positively associated with peak $\dot{V}O_{2}$ [12–14], whilst excess SED is negatively correlated with peak $\dot{V}O_{2}$ [21, 22]. However, what remains unclear is the joint association between VPA and SED as some elite junior athletes may demonstrate similar, if not more, SED than their inactive, sedentary peers [23]. Therefore, whether VPA can offset the negative effects of SED requires urgent investigation, but this cannot be done with a continued reliance on correlational statistics or the use of linear regression modelling which assumes independence between variables [23, 24]. Indeed, traditional correlational statistics are inappropriate to account for the constrained and co-dependent nature of PA data, potentially creating spurious associations.

Compositional analysis allows all movement behaviours to be expressed as a proportion of a finite period, enabling the individual, and joint, effects of movement behaviours on outcome variables to be established [16–18, 25, 26]. Consequently, compositional analysis could provide novel insights into the concomitant influences of movement behaviours, intensity, and volume on $\dot{V}O_{2\max}$. Indeed, Carson et al. [16] found that the overall movement behaviour composition explained ~38% of the variance in the peak $\dot{V}O_{2}$ of 4,169 Canadian children and adolescents (8–17 years). Despite this, when 10 minutes of time were allocated to, or removed from, MVPA, there was negligible effect on peak $\dot{V}O_{2}$, with predicted changes ranging from 0.03–0.05% [16]. This could be due, at least in part, to methodological considerations, including the estimation of peak $\dot{V}O_{2}$ from a field-based test which is likely to misrepresent true cardiorespiratory fitness [27], the pooling of data from boys and girls despite the well-established physiological differences [28], and the failure to account for maturity or training status. Furthermore, Carson et al [16] only explored the effect of changing MVPA compositions thereby combining MPA and VPA, which may potentially mask the importance of the intensity of physical activity for improving peak $\dot{V}O_{2}$ in youth. Indeed, training studies consistently show that significant improvements in absolute, and allometrically scaled, peak $\dot{V}O_{2}$ only occur when the intensity is sufficiently vigorous [10, 29]. Similarly, Gutin et al. [30] reported a stronger association between VPA and peak $\dot{V}O_{2}$ (r^2^ = 0.43, p < 0.01) than MPA (r^2^ = 0.30, p < 0.01) in adolescents, findings corroborated by both Dencker et al. [12] and Latt et al. [14] who reported that the amount of time spent in VPA explained 9.0–15.8% of the variance in peak $\dot{V}O_{2}$ in children and adolescents. However, none of these studies appropriately accounted for body mass differences, with Latt et al. [14] and Dencker et al. [13] utilising ratio scaled peak $\dot{V}O_{2}$, and Dencker et al. [12] using theoretical exponents to allometrically scale their peak $\dot{V}O_{2}$ data. By not fully accounting for body mass, heavier, more mature individuals may be penalised, creating spurious associations [15]. Hence, whether these results are a consequence of physiological pathways, or methodological inconsistencies, remains to be fully established.

Therefore, the aim of this study was to examine the independent, and interactive, effects of the five movement behaviours (SED, light intensity PA (LPA), MPA, VPA and Sleep) on $\dot{V}O_{2\max}$ in children and adolescents. The second aim was to explore the effect of baseline fitness, sex, and maturity on the predicted changes in $\dot{V}O_{2\max}$ elicited by changing PA compositions.

## 2. Methods

Ethics approval was granted by the institutional research ethics committee at Swansea University prior to the commencement of data collection and the study conformed to the Declaration of Helsinki. Before participants were accepted into this cross-sectional study, written informed parental consent and participant assent were obtained, along with all parents completing a pre-screening medical questionnaire on behalf of their child. Participants were excluded if they had known cardiovascular, metabolic, kidney, or any other disease that meant they would not have been able to complete the exercise protocol. The trained children and adolescents were all national level athletes who were part of a long-term athlete development (LTAD) program overseen by the national governing body (NGB) of their sport (Hockey, Football and Gymnastics). Untrained participants were recruited from local schools across South Wales and were not formally engaged in sport training outside of curricular physical education lessons. The final sample consisted of 237 participants, encompassing 108 trained (43 girls; age: 13.5 ± 2.1 years) and 129 untrained (51 girls; 13.8 ± 1.4 years) children and adolescents.

### 2.1 Experimental procedures

All participants were required to attend one session at which they initially had their stature and sitting height measured to the nearest 0.1 cm using a Holtain Stadiometer (Holtain, Crymych, Dyfed, UK) and their body mass measured to the nearest 0.1 kg using electronic scales (Seca 803, Seca, Chino, CA, USA). Maturity status was subsequently estimated using the equations of Mirwald et al. [31], with participants deemed pre-pubertal, pubertal, and post-pubertal if they were more than one year from, within one year of, or more than one year post peak height velocity (PHV), respectively.

$\dot{V}O_{2\max}$ was assessed using an incremental ramp test to volitional exhaustion on a cycle ergometer (Lode Excalibur Sport, Groningen, Netherlands) which started with a three-minute warm-up at 10 W before increasing by 20–25 W⋅min^-1^, depending on the participant’s age. All participants were instructed to maintain a cadence of 60–80 revolutions per minute (rpm) throughout the test, with volitional exhaustion defined as when participants could not maintain a cadence above 50 rpm. Inspired and expired air were measured on a breath-by-breath basis throughout the incremental ramp test using a Vyntus metabolic cart (VYAIRE medical Ltd, Mettawa, IL, USA). Following five minutes active and ten minutes passive rest, a supramaximal validation bout was performed [32]. Specifically, participants warmed up for a further three minutes at 10 W before a step-transition to 105% of the peak power achieved during the incremental ramp test. Participants were instructed to maintain a cadence above 50 rpm for as long as possible, with gas exchange measured continuously on a breath-by-breath basis throughout the exercise bout.

Participant’s habitual physical activity was subsequently measured for seven consecutive days using a ActiGraph GT3X (ActiGraph, Pensacola, Florida, USA) worn on the right hip, sampling at 100 Hz. Children and adolescents also completed a seven-day log to detail periods when the monitor was removed, waking time and time going to bed, to minimise the misclassification of non-wear time as sedentary time or Sleep.

### 2.2 Data analyses

The raw breath-by-breath $\dot{V}O_{2}$ data from both the $\dot{V}O_{2\max}$ and supramaximal bout were averaged into 10-second bins, with the $\dot{V}O_{2\max}$ defined as the highest 10-second moving average during the ramp incremental *or* the supramaximal test. To aid comparisons between sex, maturity, and training sub-groups, $\dot{V}O_{2\max}$ was allometrically scaled (scaled $\dot{V}O_{2\max}$) to account for body mass differences between participants [6, 32]. Evenson et al. [33] cut-points were utilised to determine the time spent in each PA intensity which have been shown to be the reliable for children and adolescents [34]. Sleep time and efficiency were calculated using the algorithms of Sadeh et al. [35]. Wear-time criteria was set as ≥ 8 hours on any three days. Using the Evenson et al. [33] cut-points, sleep algorithms, and wear-time, each day was expressed as a five-part movement composition (SED, LPA, MPA, VPA, Sleep) and linear predictive models were employed to predict changes in $\dot{V}O_{2\max}$. The smallest worthwhile change (SWC) in $\dot{V}O_{2\max}$ (l⋅min^-1^) and scaled $\dot{V}O_{2\max}$ (ml⋅kg^-b^⋅min^-1^) was calculated for each sex, maturity and training sub-group using the formula 0.2*group SD [36]. The SWC was then subsequently presented as a percentage of the group mean to aid comparisons between all sub-groups.

All compositional analyses were conducted in R (http://cran.r-project.org) using the compositions package (version 1.40–2) and its dependencies [25]. Compositional geometric means were computed to indicate the proportion of time spent in each PA behaviour or Sleep each day, by expressing each behaviour, after normalisation, as a proportion of the total time [18, 25]. Variance matrices were calculated to provide an indication as to the dispersion and co-dependency of movement behaviours and were calculated by measuring the variance between pair-wise log ratios [25, 26]. Specifically, a ratio tending towards zero indicates high co-dependency, with the numbers further from zero indicating less co-dependency. Sequential linear regression models were created by rotating each of the five behaviours via isometric log ratio (ILR) transformations to examine the relative effect of all movement behaviours on the $\dot{V}O_{2\max}$ and scaled $\dot{V}O_{2\max}$ [18, 25]. The first coefficient and its *p* value were reported for each rotation to determine whether the individual movement behaviour was associated with the outcome variable relative to the other movement behaviours, and its relative significance. Additionally, the overall model significance (*p* value) and R^2^ value were reported to gain an insight into the variance explained by the overall movement composition. All movement behaviours were also sequentially mapped against each other, producing ternary heat maps displaying the predicted absolute and scaled $\dot{V}O_{2\max}$ for each sex, training, and maturity group. Finally, change matrices were conducted to predict the change in absolute and scaled $\dot{V}O_{2\max}$ by systematically reallocating 10 minutes from one movement behaviour to another [18, 25, 26]. All predictive changes were presented as a percentage change relative to the compositional mean, with significant changes identified as any change greater than the SWC (%).

### 2.3 Statistical analyses

All traditional statistical analyses were conducted in SPSS version 26 (IBM, Portsmouth, UK), with significance accepted as p < 0.05. Between group differences in anthropometric characteristics and absolute and scaled $\dot{V}O_{2\max}$ were assessed using a MANOVA, with post-hoc tests with Bonferroni correction applied to identify the specific location of significant differences as appropriate.

## 3. Results

Of the original 237 participants, 61 were excluded for failing to meet the wear-time criteria, therefore 84 trained (40 girls) and 92 untrained (44 girls) children and adolescents were included in the final analyses. There were no significant differences in the anthropometrics of those included and excluded (p > 0.05). Post-pubertal adolescents were significantly older, taller, heavier, and more mature than the pubertal or pre-pubertal children (p < 0.01), with significant differences in the same parameters also evident between pubertal adolescents and pre-pubertal children (p < 0.05, Table 1). The trained children and adolescents were taller (*F*_(1,175)_ = 12.7, p < 0.01) and had a higher $\dot{V}O_{2\max}$ (l⋅min^-1^) than their untrained counterparts (*F*_(1, 175)_ = 15.3, p < 0.01), which persisted even after allometric scaling (*F*_(1,175)_ = 18.7, p < 0.01). Overall, boys had a higher absolute and scaled $\dot{V}O_{2\max}$ than their female counterparts, irrespective of training or maturity status (*F*_(1,175)_ = 19.7, p < 0.01). $\dot{V}O_{2\max}$ increased with maturity, irrespective of sex and training status (*F*_(1,175)_ = 16.2, p < 0.01), but there was no significant difference between any maturity group for scaled $\dot{V}O_{2\max}$. There were no significant training, sex, or maturity interactions for any anthropometric variable or $\dot{V}O_{2\max}$, regardless of how it was expressed.

**Table 1:** Participant descriptives.

Training Group	Maturity	Sex	Age (years)	Stature (cm)	Body Mass (kg)	BMI (kg∙m^-2^)	Maturity Offset (years)	$\dot{V}O_{2\max}$ (l⋅min^-1^)	Scaled $\dot{V}O_{2\max}$ (ml⋅kg^-^^b^⋅min^-1^)
Trained (n = 84)	Pre-Pubertal (n = 34)	Boys (n = 20)	11.8 ± 1.0	146.6 ± 7.5	38.5 ± 7.2	17.8 ± 2.2	-2.27 ± 0.69	2.06 ± 0.37 *	194.1 ± 22.2 *
		Girls (n = 14)	11.4 ± 1.5	151.0 ± 14.2	45.5 ± 13.7	19.4 ± 2.7	-2.37 ± 1.16	1.89 ± 0.46	153.9 ± 23.7
	Pubertal (n = 30)	Boys (n = 14)	14.1 ± 1.1 ^a^	167.9 ± 8.9 ^a^	53.3 ± 5.8 ^a^	18.9 ± 1.3	+0.01 ± 0.43 ^a^	2.77 ± 0.43 * ^a^	185.7 ± 31.6 *
		Girls (n = 16)	14.4 ± 1.3 ^a^	164.6 ± 4.4 ^a^	56.4 ± 8.1 ^a^	20.7 ± 2.4	+0.02 ± 0.57 ^a^	2.16 ± 0.27 ^a^	152.5 ± 15.5
	Post-Pubertal (n = 20)	Boys (n = 10)	16.2 ± 1.4 ^a^ ^b^	178.9 ± 6.9 ^a^ ^b^	65.2 ± 6.1 ^a^ ^b^	20.4 ± 1.9	+1.96 ± 0.57 ^a^ ^b^	3.24 ± 0.71 * ^a^ ^b^	205.5 ± 34.0 *
		Girls (n = 10)	15.8 ± 1.0 ^a^ ^b^	165.8 ± 5.7 ^a^ ^b^	58.9 ± 7.9 ^a^ ^b^	21.5 ± 3.7	+1.91 ± 0.32 ^a^ ^b^	2.30 ± 0.45 ^a^ ^b^	145.7 ± 31.2
Untrained(n = 92)	Pre-Pubertal (n = 22)	Boys (n = 12)	12.3 ± 1.7	151.5 ± 8.1	44.3 ± 10.2 ^#^	19.2 ± 3.1 ^#^	-1.94 ± 0.94	1.94 ± 0.29 ^#^ *	142.6 ± 34.7 ^#^ *
		Girls (n = 10)	12.1 ± 0.7	150.0 ± 10.9	44.9 ± 9.7 ^#^	20.0 ± 1.4 ^#^	-1.12 ± 0.12	1.35 ± 0.33 ^#^	123.9 ± 25.6 ^#^
	Pubertal (n = 40)	Boys (n = 26)	14.1 ± 0.9 ^a^	164.8 ± 8.2 ^a^	57.1 ± 11.2 ^#^ ^a^	20.9 ± 3.6 ^#^	-0.04 ± 0.66 ^a^	2.31 ± 0.47 ^#^ * ^a^	159.5 ± 34.5 ^#^ *
		Girls (n = 14)	13.1 ± 1.0 ^a^	155.8 ± 9.3 ^a^	49.4 ± 11.3 ^#^ ^a^	20.6 ± 3.4 ^#^	+0.13 ± 0.38 ^a^	1.65 ± 20.8 ^#^ ^a^	130.9 ± 20.8 ^#^
	Post-Pubertal (n = 30)	Boys (n = 10)	15.3 ± 0.3 ^a^ ^b^	172.0 ± 5.9 ^a^ ^b^	70.4 ± 14.1 ^#^ ^a^ ^b^	23.7 ± 3.7 ^#^	+1.66 ± 0.69 ^a^ ^b^	2.91 ± 0.62 ^#^ * ^a^ ^b^	166.1 ± 22.6 ^#^ *
		Girls (n = 20)	14.9 ± 0.7 ^a^ ^b^	162.3 ± 7.6 ^a^ ^b^	56.6 ± 10.2 ^#^ ^a^ ^b^	21.6 ± 3.0 ^#^	+2.10 ± 0.62 ^a^ ^b^	1.86 ± 0.38 ^#^ ^a^ ^b^	143.8 ± 34.2 ^#^

All values presented as mean ± standard deviation. BMI = Body Mass Index.

^#^ highlights a significant difference between training groups of the same sex and maturity.

^a^ significantly different compared to pre-pubertal children

^b^ Significantly different compared to pubertal adolescents.

*significant difference between boys and girls of the same training and maturity group.

### 3.1 Physical activity composition description

In the trained participants, the geometric means highlight that the largest portion of the day was spent in SED (41.2%), followed by Sleep (39.2%), with VPA only accounting for 1.6% of the day (Table 2). Similarly, untrained children spent the longest period of the day in SED (44.1%) and Sleep (40.0%), with VPA making up just 1.3% of the day. Trained athletes completed more LPA (*F*_(1.175)_ = 38.1, p < 0.01) and VPA (*F*_(1,175)_ = 18.6, p < 0.01), but spent significantly less time in Sleep (*F*_(1,175)_ = 3.8, p = 0.05) compared to untrained participants, irrespective of sex or maturity. LPA and Sleep, and SED and LPA, demonstrated the smallest variation and therefore highest co-dependency, whereas VPA had the largest pair-wise log ratio variances compared to all other PA behaviours, indicating less co-dependency (Table 3). The ILR model revealed that the overall PA composition significantly predicted both $\dot{V}O_{2\max}$ and scaled $\dot{V}O_{2\max}$ (Table 4), explaining 48.7% and 37.7%, respectively. However, when individual movements were considered in isolation, the only significant predictor of scaled $\dot{V}O_{2\max}$ was VPA (Y_VPA_ = 6.91, p < 0.02), with no significant individual associations evident for absolute $\dot{V}O_{2\max}$ (Table 4).

**Table 2:** Geometric means for the whole sample.

Trained Athletes			
	Overall Mean (minutes⋅day^-1^)	Geometric Mean (minutes⋅day^-1^)	% of 24 hours
SED	515.6	594.4	41.2
LPA	186.4	214.9	14.9
MPA	37.4	43.1	3.0
VPA	19.9	22.9	1.6
Sleep	489.7	564.5	39.2
Untrained Controls			
SED	517.2 *	634.5 *	44.1 *
LPA	134.4	164.9	11.5
MPA	37.9	46.5	3.2
VPA	15.2 *	18.6 *	1.3 *
Sleep	469.7	575.5	40.0

SED = Sedentary Time, LPA = Light Physical Activity, MPA = Moderate Physical Activity, VPA = Vigorous Physical Activity

*Indicates significant difference between training groups

**Table 3:** Pair-wise log ratio variation matrix in the full sample.

	SED	LPA	MPA	VPA	Sleep
SED	-	-0.018	-0.029	-0.053	0.023
LPA	-0.018	-	-0.039	-0.042	-0.016
MPA	-0.029	-0.039	-	-0.020	-0.019
VPA	-0.053	-0.042	0.020	-	-0.045
Sleep	0.023	-0.016	-0.019	-0.045	-

SED = Sedentary Time, LPA = Light Physical Activity, MPA = Moderate Physical Activity, VPA = Vigorous Physical Activity.

**Table 4:** Model ILR parameters for $\dot{V}O_{2\max}$ and scaled $\dot{V}O_{2\max}$.

	Model p value	Model R^2^	Y_SED_	p	Y_LPA_	p	Y_MPA_	p	Y_VPA_	p	Y_Sleep_	p
$\dot{V}O_{2\max}$ (l⋅min^-1^)	< 0.001 *	0.486	< 0.001	0.993	-0.070	0.112	0.041	0.483	0.029	0.529	< 0.001	0.997
Scaled $\dot{V}O_{2\max}$ (ml⋅kg-^b^⋅min^-1^)	< 0.001 *	0.377	-0.627	0.865	2.390	0.396	-5.606	0.136	6.914	0.019*	1.709	0.668

All models were covaried for training status, sex and maturity.

* indicates a significant predictor of outcome variable. ILR = Isometric Log Ratios, SED = Sedentary Time, LPA = Light Physical Activity, MPA = Moderate Physical Activity, VPA = Vigorous Physical Activity.

### 3.2 Impact of PA composition on $\dot{V}O_{2\max}$

Compositions with 10 minutes difference in SED, LPA, MPA, or Sleep had a minimal effect on absolute or scaled $\dot{V}O_{2\max}$ in trained children and adolescents (Table 5), with all changes smaller than the SWC (S1 Table), irrespective of training, sex, or maturity status. However, when co-varying for sex, maturity, and training status, compositions with 10 minutes more time in VPA (> 27.5 mins⋅day^-1^) compared to the average 17.5 mins⋅day^-1^ were associated with a +2.9% - 11.1% higher absolute and scaled $\dot{V}O_{2\max}$ and compositions with less than 10 mins⋅day^-1^ of VPA were associated with a reduced absolute and scaled $\dot{V}O_{2\max}$ (-4.6% - 24.4%). The proportion of SED, LPA, and Sleep had little impact on absolute and scaled $\dot{V}O_{2\max}$ (0.01–1.98%). Consequently, VPA was the most influential PA behaviour for absolute, and scaled $\dot{V}O_{2\max}$, irrespective of training status and sex, but the influence of PA behaviours was less clear in pubertal and post-pubertal adolescents (S1 and S2 Figs).

**Table 5:** Change matrices of reallocating 10 minutes from the behaviour in columns to the behaviour in the rows on $\dot{V}O_{2\max}$ (l⋅min^-1^) and scaled $\dot{V}O_{2\max}$ (ml⋅kg^-b^⋅min^-1^) in trained children and adolescents, presented as percentage change.

$\dot{V}O_{2\max}$						Scaled $\dot{V}O_{2\max}$					
Pre-Pubertal Boys											
	SED	LPA	MPA	VPA	Sleep		SED	LPA	MPA	VPA	Sleep
SED	-	0.31	-1.11	-1.59	0.01	SED	-	0.11	1.78	-4.58 *	-0.06
LPA	-0.31	-	-1.42	-1.90	-0.31	LPA	-0.11	-	1.67	-4.69 *	-0.16
MPA	0.87	1.12	-	-0.72	0.87	MPA	-1.39	-1.27	-	-5.97 *	-1.45
VPA	1.01	1.33	-0.10	-	1.01	VPA	2.91 *	3.02 *	4.69 *	-	2.85 *
Sleep	-0.01	0.32	-1.11	-1.59	-	Sleep	0.05	0.17	1.84	-4.52 *	-
Pre-Pubertal Girls											
	SED	LPA	MPA	VPA	Sleep		SED	LPA	MPA	VPA	Sleep
SED	-	0.49	-1.80	-3.21	0.01	SED	-	0.15	2.49	-7.96 *	-0.06
LPA	-0.46	-	-2.25	-3.67	0.49	LPA	-0.14	-	2.35	-8.10 *	0.12
MPA	1.35	1.84	-	-1.86	1.35	MPA	-1.88	-1.72	-	-9.83 *	-1.94
VPA	1.73	2.21	-0.07	-	1.73	VPA	4.29 *	4.45 *	6.79 *	-	4.23 *
Sleep	-0.01	0.49	-1.80	-3.21	-	Sleep	0.06	0.22	2.56	-7.90 *	-
Pubertal Boys											
	SED	LPA	MPA	VPA	Sleep		SED	LPA	MPA	VPA	Sleep
SED	-	0.33	-0.87	-1.28	0.01	SED	-	0.15	1.70	-4.47 *	-0.05
LPA	-0.31	-	-1.18	-1.59	-0.31	LPA	-0.13	-	1.56	-4.61 *	-0.19
MPA	0.69	1.02	-	-0.59	0.69	MPA	-1.34	-1.19	-	-5.81 *	-1.39
VPA	0.82	1.15	-0.06	-	0.82	VPA	2.86	3.00	4.56 *	-	2.80
Sleep	-0.01	0.33	-0.87	-1.28	-	Sleep	0.05	0.20	1.75	-4.41 *	-
Pubertal Girls											
	SED	LPA	MPA	VPA	Sleep		SED	LPA	MPA	VPA	Sleep
SED	-	0.45	-1.79	-7.60 *	0.01	SED	-	0.19	3.24 *	-24.4 *	-0.07
LPA	-0.43	-	-1.34	-8.03 *	-0.43	LPA	-0.18	-	3.46 *	-24.6 *	-0.24
MPA	1.27	1.72	-	-6.33 *	1.27	MPA	-2.28 *	-2.10 *	-	-26.7 *	-2.35 *
VPA	1.98	2.44	0.19	-	1.98	VPA	6.38 *	6.57 *	9.62 *	-	6.31 *
Sleep	-0.01	0.45	-1.80	-7.60 *		Sleep	0.07	0.25	3.30 *	-24.3 *	-
Post-Pubertal Boys											
	SED	LPA	MPA	VPA	Sleep		SED	LPA	MPA	VPA	Sleep
SED	-	0.32	-0.96	-1.11	0.01	SED	-	0.16	2.03	-4.21 *	-0.05
LPA	-0.30	-	-1.26	-1.41	-0.30	LPA	-0.15	-	1.88	-4.36 *	-0.20
MPA	0.71	1.03	-	0.40	0.72	MPA	-1.51	-1.35	-	-5.72 *	-1.56
VPA	0.71	1.03	-0.25	-	0.71	VPA	2.68	2.84	4.71 *	-	2.63
Sleep	-0.01	0.32	-0.96	-1.12	-	Sleep	0.05	0.21	2.08	-4.16 *	-
Post-Pubertal Girls											
	SED	LPA	MPA	VPA	Sleep		SED	LPA	MPA	VPA	Sleep
SED	-	0.41	-1.25	-1.89	0.01	SED	-	0.19	2.50	-6.75 *	-0.06
LPA	-0.38	-	-1.88	-2.27	-0.38	LPA	-0.18	-	2.32	-6.93 *	0.23
MPA	0.92	1.32	-	-0.97	0.90	MPA	-1.84	-1.65	-	-8.59 *	-1.90
VPA	1.05	1.46	-2.27	-	1.05	VPA	3.77	3.96	6.27 *	-	3.71
Sleep	-0.01	0.40	-0.97	-1.89	-	Sleep	0.06	0.25	2.48	-6.69 *	-

SED = Sedentary time, LPA = Light Intensity Physical Activity, MPA = Moderate Physical Activity, VPA = Vigorous Physical Activity. All figures presented as percentage change with

* indicating a change above the Smallest Worthwhile Change (%).

Contrastingly, in pre-pubertal untrained children, compositions with 10 minutes less VPA (-5.2 mins∙day^-1^) compared to the average 15.2 mins∙day^-1^ were associated with a decrease in $\dot{V}O_{2\max}$ of between 3.2–10.5% (Table 6). Additionally, compositions with less LPA, but more MPA or VPA, were associated with an increase in $\dot{V}O_{2\max}$ between 5.2% and 5.8% in pre-pubertal untrained girls.

**Table 6:** Change matrices of reallocating 10 minutes from the behaviour in columns to the behaviour in the rows on $\dot{V}O_{2\max}$ (l⋅min^-1^) and scaled $\dot{V}O_{2\max}$ (ml⋅kg^-b^⋅min^-1^) in untrained children and adolescents, presented as percentage change.

$\dot{V}O_{2\max}$						Scaled $\dot{V}O_{2\max}$					
Pre-Pubertal Boys											
	SED	LPA	MPA	VPA	Sleep		SED	LPA	MPA	VPA	Sleep
SED	-	0.63	-1.34	-3.17 *	0.01	SED	-	0.23	2.11	-8.93 *	-0.07
LPA	-0.59	-	-1.93	-3.75 *	-0.59	LPA	-0.22	-	1.90	-9.14 *	-0.28
MPA	1.05	1.69	-	-2.11	1.05	MPA	-1.66	-1.42	-	-10.59 *	-1.72
VPA	1.62	2.25	0.27	-	1.62	VPA	4.58	4.81	6.69 *	-	4.51
Sleep	-0.01	0.63	-1.34	-3.17		Sleep	0.07	0.30	2.18	-9.15 *	-
Pre-Pubertal Girls											
	SED	LPA	MPA	VPA	Sleep		SED	LPA	MPA	VPA	Sleep
SED	-	2.72	-3.93	-8.31 *	0.01	SED	-	0.88	5.17 *	-19.43 *	-0.09
LPA	-2.21	-	-6.14 *	-10.52 *	-2.20	LPA	-0.71	-	4.46 *	-20.13 *	-0.79
MPA	2.48	5.20 *	-	-5.84 *	2.48	MPA	-3.25	-2.36	-	-22.67 *	-3.33
VPA	2.86	5.58 *	-1.07	-	2.86	VPA	6.70 *	7.57 *	11.86 *	-	6.61 *
Sleep	-0.01	2.72	-3.94	-8.31 *		Sleep	0.06	0.96	5.24 *	-19.34 *	-
Pubertal Boys											
	SED	LPA	MPA	VPA	Sleep		SED	LPA	MPA	VPA	Sleep
SED	-	0.53	-1.12	-3.00	0.01	SED	-	0.27	2.22	-10.64 *	-0.07
LPA	-0.53	-	-1.64	-3.53	-0.53	LPA	-0.25	-	1.97	-10.89 *	-0.32
MPA	0.87	1.44	-	-2.13	0.87	MPA	-1.72	-1.45	-	-12.36 *	-1.79
VPA	1.40	1.97	0.28	-	1.40	VPA	4.96 *	5.23 *	7.18 *	-	4.89 *
Sleep	-0.01	0.57	-1.12	-3.01	-	Sleep	0.07	0.34	2.29	-10.58 *	-
Pubertal Girls											
	SED	LPA	MPA	VPA	Sleep		SED	LPA	MPA	VPA	Sleep
SED	-	0.77	-1.43	-3.85	0.01	SED	-	0.33	2.58	-12.32 *	-0.08
LPA	-0.71	-	-2.15	-4.57	-0.71	LPA	-0.30	-	2.28	-12.63 *	-0.38
MPA	1.13	1.90	-	-2.72	1.13	MPA	-2.02	-1.68	-	-14.34 *	-2.10
VPA	1.84	2.62	2.72	-	1.84	VPA	5.90 *	6.22 *	4.90 *	-	5.81 *
Sleep	-0.01	0.77	-1.44	-3.86		Sleep	0.08	0.41	2.66	-12.25 *	-
Post-Pubertal Boys											
	SED	LPA	MPA	VPA	Sleep		SED	LPA	MPA	VPA	Sleep
SED	-	0.62	-1.23	-2.30	0.01	SED	-	0.32	2.67	-8.89 *	-0.06
LPA	-0.56	-	-1.79	-2.86	-0.56	LPA	-0.29	-	2.38	-9.18 *	-0.35
MPA	0.89	1.51	-	-1.41	0.89	MPA	-1.93	-1.60	-	-10.82 *	-1.99
VPA	1.12	1.74	-0.11	-	1.12	VPA	4.34 *	4.66 *	7.00 *	-	4.27 *
Sleep	-0.01	0.62	-1.23	-2.30	-	Sleep	0.06	0.38	2.73 *	-8.83 *	-
Post-Pubertal Girls											
	SED	LPA	MPA	VPA	Sleep		SED	LPA	MPA	VPA	Sleep
SED	-	0.44	-1.54	-3.55	0.01	SED	-	0.02	3.10	-12.70 *	-0.08
LPA	-0.42	-	-1.96	-3.96	-0.41	LPA	-0.19	-	2.91	-12.89 *	-0.27
MPA	1.14	1.57	-	-2.41	1.14	MPA	-2.28	-2.07	-	-14.98 *	-2.36
VPA	1.59	2.02	1.59	-	1.59	VPA	5.70 *	5.91 *	5.68 *	-	5.63 *
Sleep	-0.01	0.44	-1.54	-3.54	-	Sleep	0.08	0.28	3.17	-12.62 *	-

SED = Sedentary time, LPA = Light Physical Activity, MPA = Moderate Physical Activity, VPA = Vigorous Physical Activity. All figures presented as percentage change with * indicating a change greater than the Smallest Worthwhile Change (%).

Scaled $\dot{V}O_{2\max}$ significantly decreased, irrespective of sex, maturity, or training status, when 10 minutes of VPA was reallocated to any other behaviour (Tables 5 and 6). Moreover, scaled $\dot{V}O_{2\max}$ tended to increase when time spent in other movement behaviours was reallocated to VPA. In isolation, both SED and Sleep were not significant predictors of absolute or scaled $\dot{V}O_{2\max}$ (Table 4) and displacing SED with any other behaviour had a negligible effect on $\dot{V}O_{2\max}$ (0.01–1.98%; Tables 5 and 6) unless it was displaced with VPA where it was associated with an increase of 2.68–6.38% in scaled $\dot{V}O_{2\max}$ only. Similarly, compositions with 10 minutes less Sleep and 10 minutes more VPA were associated with a 2.85–6.31% greater scaled $\dot{V}O_{2\max}$, irrespective of sex, training, and maturation. The effects of reallocating time to LPA, SED, or Sleep on $\dot{V}O_{2\max}$ were negligible, regardless of how $\dot{V}O_{2\max}$ was expressed and irrespective of sex, maturity, or training status.

## 4. Discussion

This is the first study to examine the inter-related effects of various movement behaviours (SED, LPA, MPA, VPA and Sleep), using a five-part compositional analysis, on absolute and scaled $\dot{V}O_{2\max}$ in trained and untrained children and adolescents. The main findings of the present study were that allocating time to, and removing time from, VPA significantly increased and decreased scaled $\dot{V}O_{2\max}$, respectively, regardless of sex, training, or maturity status. Moreover, this study suggests that VPA is potentially 2.4–4.7% more potent in eliciting an improvement in VO_2max_ over a 10-minute period in children and adolescents. These findings therefore highlight that intensity of PA may be of paramount importance in determining $\dot{V}O_{2\max}$, especially in girls.

Engaging in 10 minutes more VPA, irrespective of which behaviour it displaces, significantly increases both absolute and scaled $\dot{V}O_{2\max}$, regardless of training status. Moreover, of importance, untrained children were predicted to have a larger magnitude of change for the same 10-minute reallocation, in accord with the review of McNarry & Jones [8] which concluded that baseline fitness significantly impacts the magnitude of change experienced following a given stimulus. Furthermore, Mahon [37] reported that 52% of the inter-individual variation in participant’s responses to a training stimulus can be explained by baseline peak $\dot{V}O_{2}$. The findings of the current study are, however, discordant with Carson et al. [16] who reported no significant differences when reallocating time to, or from, any movement behaviour. Such discrepancies may be explained by the use of a proxy measure of $\dot{V}O_{2\max}$ and not accounting for maturation or training status in the earlier study, which are critical when assessing cardiorespiratory fitness in children and adolescents [10, 11, 27].

The present study supports the notion that children and adolescents may require a vigorous stimulus to significantly improve absolute and scaled $\dot{V}O_{2\max}$ [8, 10, 12–14]. Whilst increasing levels of VPA is relatively time efficient, addressing a commonly cited barrier to physical activity [38], it is pertinent to note that the current findings suggest children and adolescents may need to increase their time spent in VPA by over 50%. Considering the limited success at increasing PA in many interventions to date [38, 39], and the small magnitude of increases in VPA reported even in those considered successful [40], the current findings highlight the need to drastically change our approach to PA promotion. These findings could be speculated to support the contention suggested by many authors that HIIT may represent an important public health intervention tool [29, 41].

Dencker et al. [12] reported weak, but significant, correlations between VPA and peak $\dot{V}O_{2}$ (r^2^ = 0.32) and scaled peak $\dot{V}O_{2}$ (r^2^ = 0.27). Furthermore, a recent review of the relationship between PA and peak $\dot{V}O_{2}$ in youth concluded that, despite decades of research, there was still no consensus [11]. These equivocal findings may be related to a reliance on techniques that fail to account for the inter-related and inherently constrained nature of PA behaviours, leading to spurious conclusions [18, 25, 26]. Moreover, the reliance on ratio scaled peak $\dot{V}O_{2}$ potentially creates erroneous associations [15, 42]. Of note, in the present study when $\dot{V}O_{2\max}$ was allometrically scaled by body mass, the overall PA composition explained ~11% less variance compared to absolute $\dot{V}O_{2\max}$. This may be due, at least in part, to physically active children having a higher lean body mass (LBM) than their sedentary counterparts [43], indicating that differences in body composition may also be critical when determining the effect of re-allocating PA. Nevertheless, the PA composition still explained 37.7% of the variance in allometrically scaled $\dot{V}O_{2\max}$, demonstrating the powerful influence of habitual PA on aerobic fitness.

The finding that allocating time to MPA decreased scaled $\dot{V}O_{2\max}$ was surprising. These associations could be to the high amount of VPA undertaken by the trained group within this study and, consequently, if time is removed and replaced with MPA, it will have a negative impact on $\dot{V}O_{2\max}$. Future research is required to explore the interaction of MPA and VPA in other athletic populations to confirm this hypothesis. Of note, the fixed time reallocation used within compositional analysis studies to date [18, 20], and in the present study, may over-estimate the magnitude of change in a given variable. More specifically, a 10-minute change in VPA constituted a ~50% increase in VPA within the current sample but the same 10-minute reallocation only represented a 1.9% increase in SED time. Therefore, a greater insight into the independent, and interactive, effects of movement behaviours on $\dot{V}O_{2\max}$ may be gained by investigating the effects of the same percentage change in movement behaviours on $\dot{V}O_{2\max}$. Nevertheless, evidence is emerging that the intensity of PA may be critical in improving both performance and health-related parameters in paediatric populations [18, 20, 25] and thus VPA should be encouraged, as opposed to MPA, to engender the greatest long-term health benefits.

Sedentary time in isolation was not a significant predictor of either absolute or scaled $\dot{V}O_{2\max}$ in children and adolescents within the current study, irrespective of sex, maturation, or training status. This is in direct contrast to the growing body of literature suggesting that excess sedentary time could lead to a decreased $\dot{V}O_{2\max}$ [21, 22] and suggests that instead of SED being the problem *per se*, it is potentially the PA behaviour it replaces that is influential. More specifically, significant increases in $\dot{V}O_{2\max}$ were only associated with compositions where the amount of time spent sedentary decreased and was replaced with VPA. However, whilst displacing SED with LPA and MPA were not associated with an increased $\dot{V}O_{2\max}$, if these behavioural changes were introduced as part of a wider health initiative, they could still contribute to improving the health of the nation.

Future research should seek to implement targeted interventions informed by compositional analyses to ascertain the required duration needed to elicit the changes predicted. This is of particular importance as a plethora of research has investigated the influence of different training methodologies on both absolute and scaled $\dot{V}O_{2\max}$, with their effectiveness being reviewed elsewhere [8, 10]. One major issue with many paediatric training studies to date is the lack of accounting for changes in habitual PA levels across the intervention period, and this could help explain the equivocal findings of some intervention types [10, 43].

Whilst there are numerous strengths associated with this study, such as the use of a novel five-part compositional analysis approach, allometrically scaling $\dot{V}O_{2\max}$, and accounting for training, maturity and sex differences, there are limitations which must be acknowledged. Firstly, a relatively low wear-time criteria was set of any three days with at least eight hours of wear-time; a more stringent wear-time criteria could potentially influence the relationships established between PA metrics and $\dot{V}O_{2\max}$. Nevertheless, this wear-time has been validated in a paediatric population [34] and was used to maximise participant inclusion within the study. Secondly, the cross-sectional study does not allow the duration over which the habitual changes need to be maintained to observe the associated changes to be elucidated. Thirdly, the small sample size compared to other studies of this type [18, 25] and the representativity of this population needs to be considered when interpreting the results of this study. Finally, the applicability of cycle derived $\dot{V}O_{2\max}$ to habitual PA levels is contentious, and therefore future research should endeavour to establish $\dot{V}O_{2\max}$ using treadmills to maximise specificity, and to establish whether these findings persist.

## 5. Conclusion

In conclusion, the proportion of time VPA is a significant predictor of scaled $\dot{V}O_{2\max}$ in children and adolescents, independent of training, sex, or maturity status and even when the proportion of time spent in other behaviours is considered. Moreover, reallocating time from VPA in pre-pubertal children predicts a reduced absolute $\dot{V}O_{2\max}$, potentially highlighting the importance of promoting VPA in pre-pubertal children. Future research should seek to establish the duration of targeted PA interventions needed to elicit the significant changes predicted from compositional analyses and report the individual levels of MPA and VPA to ascertain the relative importance of VPA for current, and future, health in children and adolescents.

## Supporting information

S1 TableSmallest worthwhile change in all sub-groups for $\dot{V}O_{2\max}$ (l⋅min-1) and allometrically scaled $\dot{V}O_{2\max}$ (ml⋅kg^-b^⋅min^-1^).SWC = Smallest Worthwhile change, SWC (%) = Smallest worthwhile change as a percentage of the individual group mean.(TIF)

S1 FigTernary heat plots for 24-hour movement behaviours and predicted $\dot{V}O_{2\max}$.Ternary heat plots of all PA behaviours with expected $\dot{V}O_{2\max}$ values for all sub-groups with a) trained athletes; b) untrained controls; c) all boys; d) all girls; e) pre-pubertal children; f) pubertal adolescents; and g) post-pubertal adolescents,(TIF)

S2 FigTernary heat plots for 24-hour movement behaviours and predicted allometrically scaled $\dot{V}O_{2\max}$.Ternary plots of all PA behaviours with expected scaled $\dot{V}O_{2\max}$ values for all sub-groups with a) trained athletes; b) untrained controls; c) all boys; d) all girls; e) pre-pubertal children; f) pubertal adolescents; and g) post-pubertal adolescent.(TIF)

S1 Data The data used to run the compositional analyses and produce the outcomes for this manuscript are included as supplementary material. (XLSX)
