# Supplementary figures and images for: The associations of physical activity, sedentary time, and sleep with V˙O2max in trained and untrained children and adolescents: A novel five-part compositional analysis
Source: PLoS One. 2023 Mar 8;18(3):e0275557. doi: 10.1371/journal.pone.0275557 (PMC9994740; doi:10.1371/journal.pone.0275557)

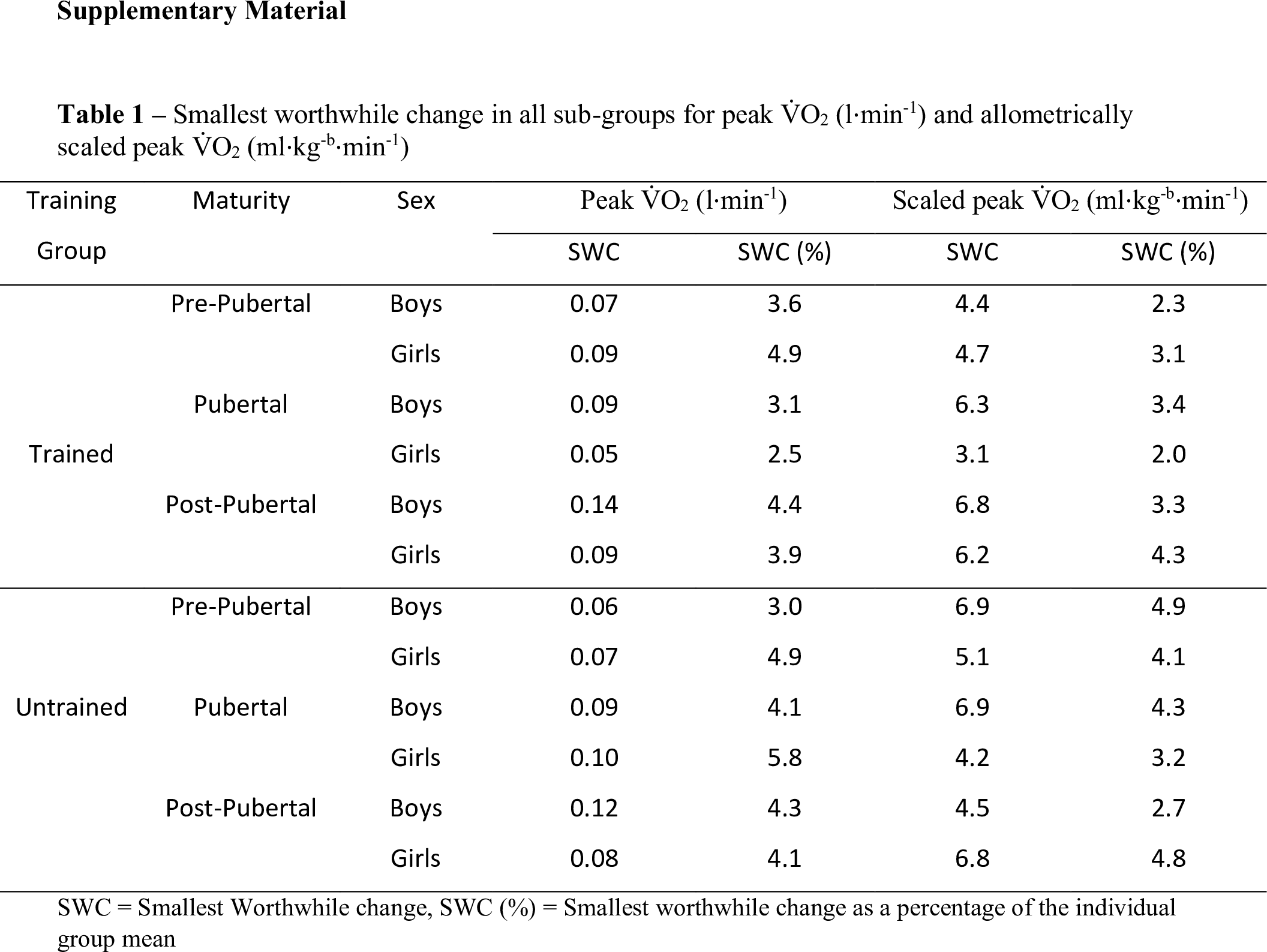

Supplement: S1 Table — SWC = Smallest Worthwhile change, SWC (%) = Smallest worthwhile change as a percentage of the individual group mean. (TIF) [file pone.0275557.s001.tif]

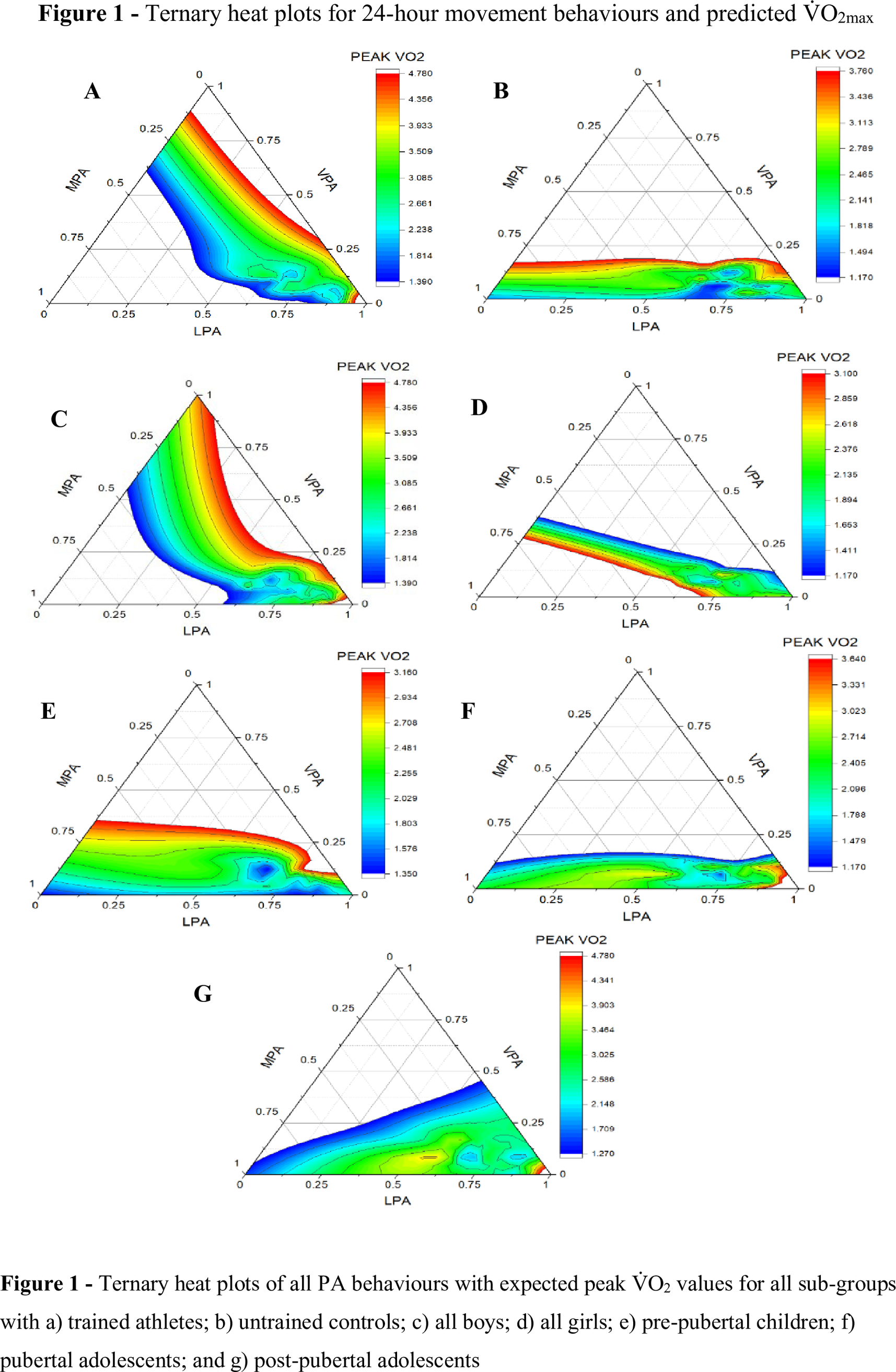

Supplement: S1 Fig — Ternary heat plots of all PA behaviours with expected V˙O2max values for all sub-groups with a) trained athletes; b) untrained controls; c) all boys; d) all girls; e) pre-pubertal children; f) pubertal adolescents; and g) post-pubertal adolescents, (TIF) [file pone.0275557.s002.tif]

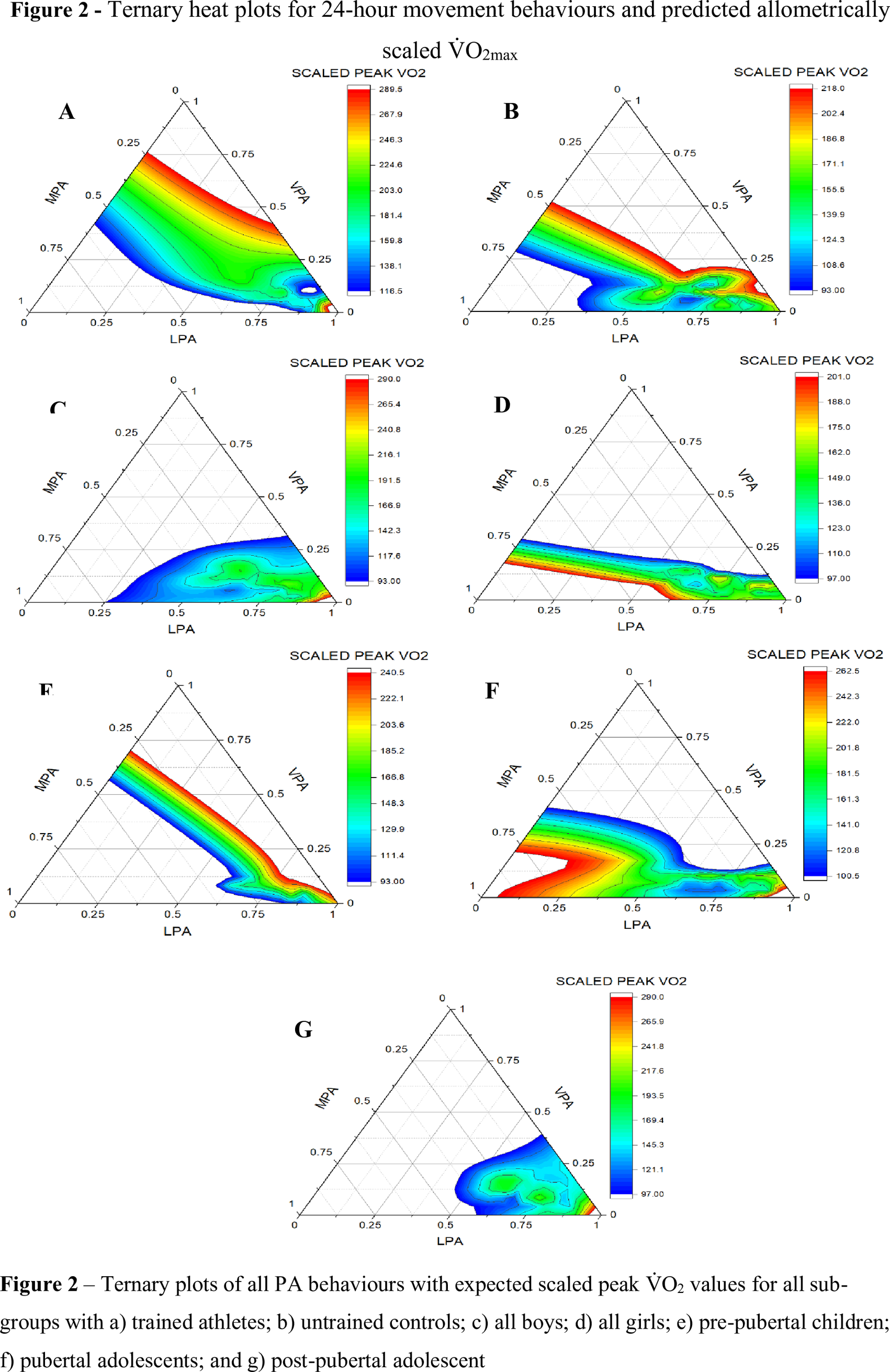

Supplement: S2 Fig — Ternary plots of all PA behaviours with expected scaled V˙O2max values for all sub-groups with a) trained athletes; b) untrained controls; c) all boys; d) all girls; e) pre-pubertal children; f) pubertal adolescents; and g) post-pubertal adolescent. (TIF) [file pone.0275557.s003.tif]
